# Concerted Influence of H_2_O and CO_2_: Moisture Exposure of Sulfide Solid Electrolyte Li_4_SnS_4_

**DOI:** 10.1021/acsomega.4c03685

**Published:** 2024-09-06

**Authors:** Yusuke Morino, Misae Otoyama, Toyoki Okumura, Kentaro Kuratani, Naoya Shibata, Daisuke Ito, Hikaru Sano

**Affiliations:** † Murata Manufacturing Co., Ltd., 1-10-1 Higashikotari, Nagaokakyo-shi, Kyoto 617-8555, Japan; ‡ National Institute of Advanced Industrial Science and Technology (AIST), 1-8-31 Midorigaoka, Ikeda, Osaka 563-8577, Japan

## Abstract

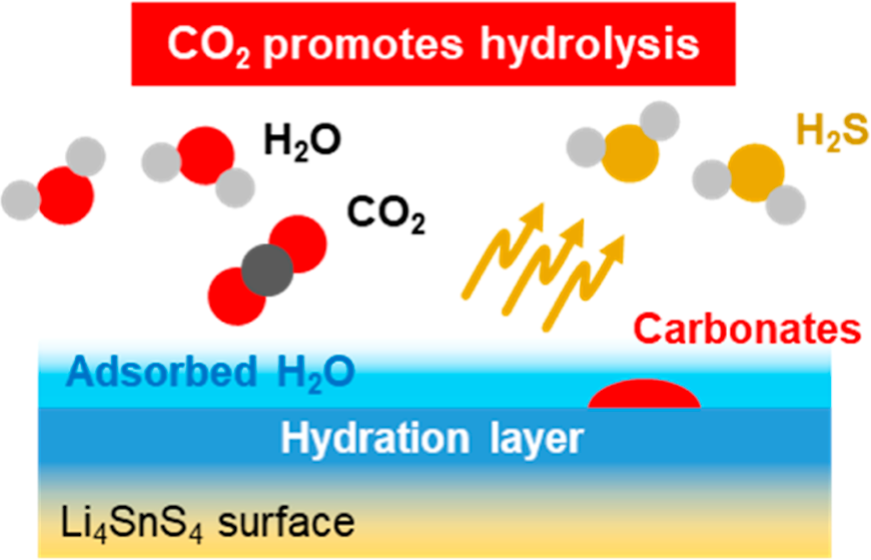

Although moisture-induced deterioration mechanisms in sulfide solid electrolytes to enhance atmospheric stability have been investigated, the additional impact of CO_2_ exposure remains unclear. This study investigated the generation of H_2_S from Li_4_SnS_4_ under H_2_O and CO_2_ exposure. Li_4_SnS_4_ was exposed to Ar gas at a dew point of 0 °C with and without 500 ppm of CO_2_, and its ion conductive properties were evaluated. Although the lithium-ion conductivity of Li_4_SnS_4_ decreased regardless of the presence of CO_2_, the amount of H_2_S generated with CO_2_ was five times higher. To elucidate the underlying mechanism, X-ray diffraction and Raman spectroscopy were used. Without CO_2_, hydrate Li_4_SnS_4_·4H_2_O formation markedly increased, whereas, with CO_2_, it increased a little. The difference revealed distinct deterioration mechanisms leading to a decrease in lithium-ion conductivity: without CO_2_, adsorbed H_2_O and Li_4_SnS_4_·4H_2_O contributed to the decrease, while with CO_2_, a weak acid dissociation reaction could reduce the thermodynamic stability of the moisture-exposed Li_4_SnS_4_ surface including Li_4_SnS_4_·4H_2_O and adsorbed H_2_O, promoting H_2_S release and carbonate formation. This was supported by the recovery of lithium-ion conductivity after vacuum heating. The concerted influence of H_2_O and CO_2_ provides valuable insights into the fundamental deterioration mechanisms in sulfide solid electrolytes that could be applied in battery manufacturing processes.

## Introduction

All-solid-state batteries using inorganic solid electrolytes (SEs) are considered promising energy devices due to their higher-rate charge/discharge capability, longer lifetime expectancy compared to that of present liquid-type lithium-ion batteries (LIBs), enhanced safety features, and wider operating temperature range. Among various types of inorganic SEs, sulfide SE materials based on Li–P–S systems are widely recognized for their excellent formability along with their high lithium ionic conductivities.^1−4^ Additionally, it has been observed that all-solid-state batteries employing sulfide SEs exhibit remarkable durability even under extreme conditions such as low- or high-temperature environments or during operation at high voltages.^1,5−9^ However, sulfide SEs possess an inherent challenge: they tend to produce toxic H_2_S gas upon exposure to humidity.^10−14^ Thus, numerous research groups are actively engaged in developing more stable alternatives.^15−20^

Recently, Li_4_SnS_4_ (LSS) has attracted attention as an SE material capable of exhibiting both good lithium ion conductivity and superior moisture durability. For example, it has been reported that hexagonal LSS synthesized via mechanical milling followed by low-temperature heat treatment generates an exceptionally small quantity of H_2_S gas (∼0.2 cm^3^ g^–1^) during exposure to temperatures ranging from 20 to 22 °C and relative humidity (R.H.) of 70% for 40 min^19^ Besides, various attempts have also been made to dope LSS with Li_3_PS_4_^20−22^ and other additives^23,24^ in order to promote enhanced lithium ionic conductivity while maintaining moisture durability. Investigating beyond material development, Kimura et al. have analyzed the mechanisms underlying the moisture durability in both hexagonal and orthorhombic LSS when exposed to humidified inert gases such as N_2_ and Ar. Interestingly, the formation of hydrate Li_4_SnS_4_·4H_2_O upon exposure to humidity followed by its recovery to an anhydrous state via heat treatment under vacuum conditions was observed.^25^ It is considered that the generation of H_2_S gas is significantly suppressed due to the thermodynamically stable hydrate Li_4_SnS_4_·4H_2_O resulting from S atoms shared between LiS_2_(H_2_O)_2_ tetrahedra and SnS_4_^4–^ tetrahedra.^25^ In response, several aqueous synthesis methods have been proposed that take advantage of this excellent moisture durability.^26−28^

Several studies have suggested that O_2_ and CO_2_, in addition to H_2_O (moisture), can influence the deteriorative processes associated with sulfide SE materials.^10−13,29^ As a matter of fact, during industrial processes such as battery manufacturing, material synthesis, and storage, the environmental atmosphere typically contains not only moisture but also a mixture of various gases. Therefore, in this study, we focused on investigating the influence of CO_2_ on moisture exposure. The LSS powder samples were exposed to moisture-controlled Ar gas flow with and without CO_2_ while monitoring the amount of H_2_S gas generation. Afterward, the samples were characterized by lithium ionic conductivity measurement using electrochemical impedance spectroscopy (EIS), X-ray diffraction (XRD), and Raman spectroscopy to elucidate the effects associated with CO_2_ during moisture exposure and to understand the corresponding deterioration mechanisms. Finally, vacuum heating was performed to verify the deterioration mechanisms and to demonstrate a recovery treatment for the successful implementation of all-solid-state batteries.

## Experimental Section

### Material and Preparation

**Figure 1:**
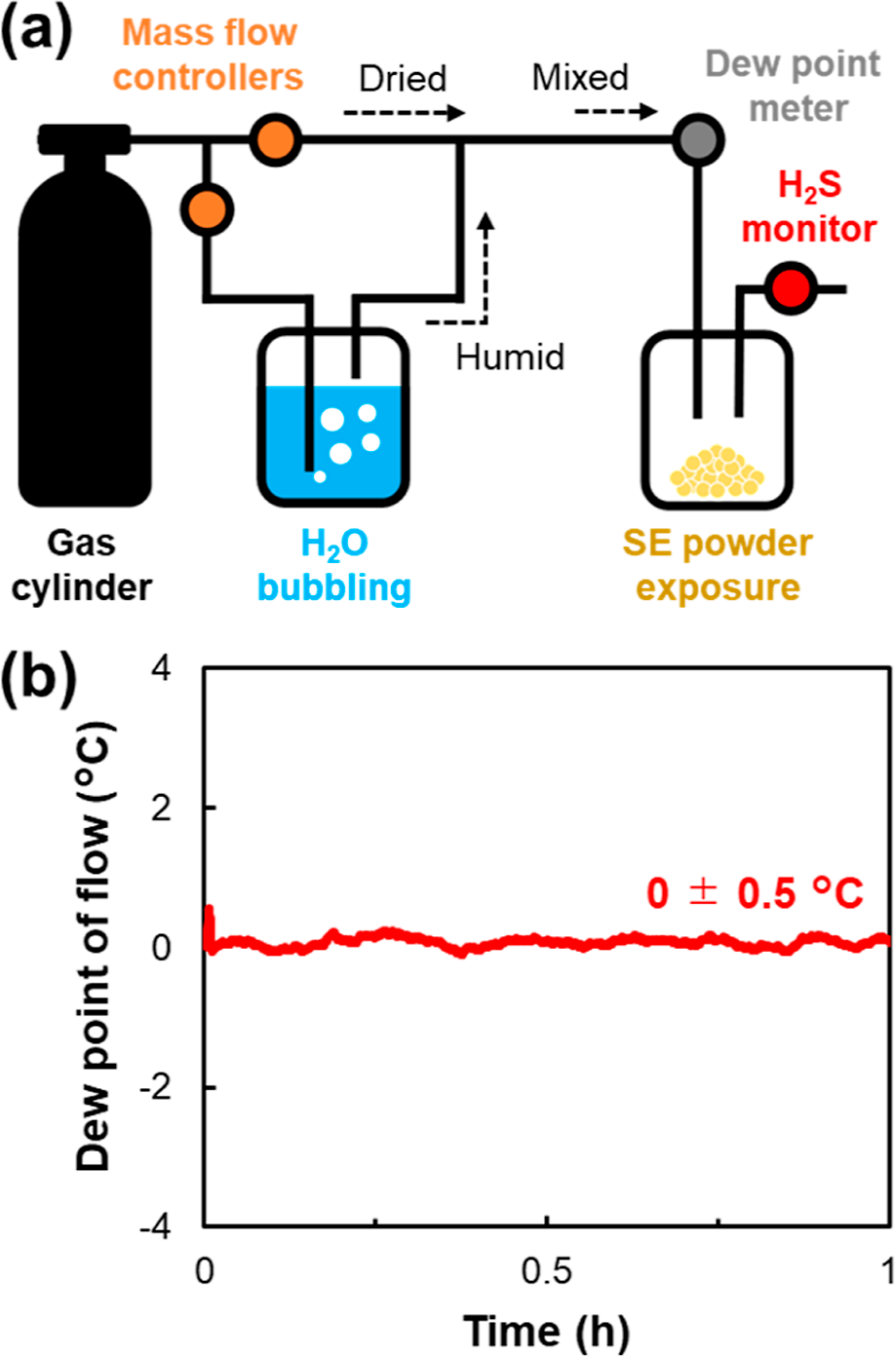
(a) System of controlled moisture exposure and H_2_S monitoring. (b) Dew point monitoring when humidity was controlled by setting the target to a dew point of 0 °C.

A sulfide SE of LSS powder was synthesized according to a previously reported procedure;^26,27^ Li_2_S, Sn, and S were introduced into ultrapure H_2_O in a molar ratio of 2:1:2. The mixture was dissolved while stirring at 80 °C for more than 12 h and then dried at 120 °C in a vacuum for 3 h to obtain a hexagonal LSS powder with an average particle size of approximately 1 μm. The controlled moisture exposure and H_2_S monitoring system (Figure 1a) was constructed based on the report by Yamada et al.^30^ A gas cylinder containing Ar (<0.1 ppm of CO_2_) or Ar + 500 ppm of CO_2_ with a dew point below −80 °C (<0.5 ppm of H_2_O) was purchased (Grade 1, Taiyo Nippon Sanso Co., Japan) and connected to the upstream end of the system. The gas line was split into two branches. In one branched line, by bubbling argon gas through water, we generated a gas that contained water vapor. The two gas lines were then united under control of each flow rate by using mass flow controllers in order to prepare moisture-controlled gas. The dew point of the moisture-controlled gas was confirmed using an in-line dew point meter as 0 °C (∼6000 ppm of H_2_O) (Figure 1b). Then, 200 mg of LSS powder was exposed to the controlled-moisture gas at a flow rate of 0.8 L min^–1^ for 1 h. A H_2_S sensor (Model RS3000, Advanced Micro Instruments, USA) was connected to the line after the exposed LSS sample to monitor the amount of H_2_S gas generated. The total amount of generated H_2_S was calculated per 1 g of SE by accumulating the concentration value. The LSS samples before exposure are labeled as “pristine”, the sample after exposure to a dew point of 0 °C without CO_2_ as “without CO_2_”, and the sample after exposure to a dew point of 0 °C with CO_2_ as “with CO_2_,” thereafter. Additionally, a recovery treatment demonstration was conducted under the same conditions as the synthesis process at 120 °C in a vacuum for 3 h.

### Lithium Ionic Conductivity

The LSS samples, 80 mg, were pelletized at 360 MPa and restrained at a pressure of 98 ppm in a zirconia cylinder with a diameter of 10 mm between two stainless-steel (SUS) electrodes. The lithium ionic conductivity was evaluated using EIS measurement with a voltage amplitude of 30 mV in a frequency range of 10^6^–10^1^ Hz at a temperature of 22 °C. Nyquist and Bode plots were used to describe the normalized impedance (measured in kΩ cm), taking into account the pellet thickness and electrode area.

### X-ray Diffraction

XRD measurements were performed using a reflective configuration system (Empyrean, Malvern PANalytical, UK) and an airtight sample holder with knife edge. The diffraction patterns were obtained in the 2θ range 10–80° with a step width of 0.1°, using Cu Kα as the X-ray source. The diffraction patterns were analyzed using the software package “The General Structure and Analysis Software II (GSAS-II)”.^31^

### Raman Spectroscopy

Raman spectroscopy measurements were conducted using a system (RAMANforce, Nanophoton, Japan) equipped with an incident green laser at a wavelength of 523 nm, which was directed through a quartz glass window in an airtight sample holder. The measurements were performed under controlled conditions to ensure that the signal-to-noise ratio of the Raman signal was within acceptable limits: The laser output was reduced to 10 μW μm^–2^, and the laser exposure time was set to 10 s to prevent damage such as desorption of adsorbed H_2_O and decomposition of SE. The Raman spectra were calibrated by fixing the Si wafer peak at 520 cm^–1^ and by normalizing the peak intensity of the Sn–S vibration at 354 cm^–1^ of the SnS_4_^4–^ unit.

## Results and Discussion

**Figure 2:**
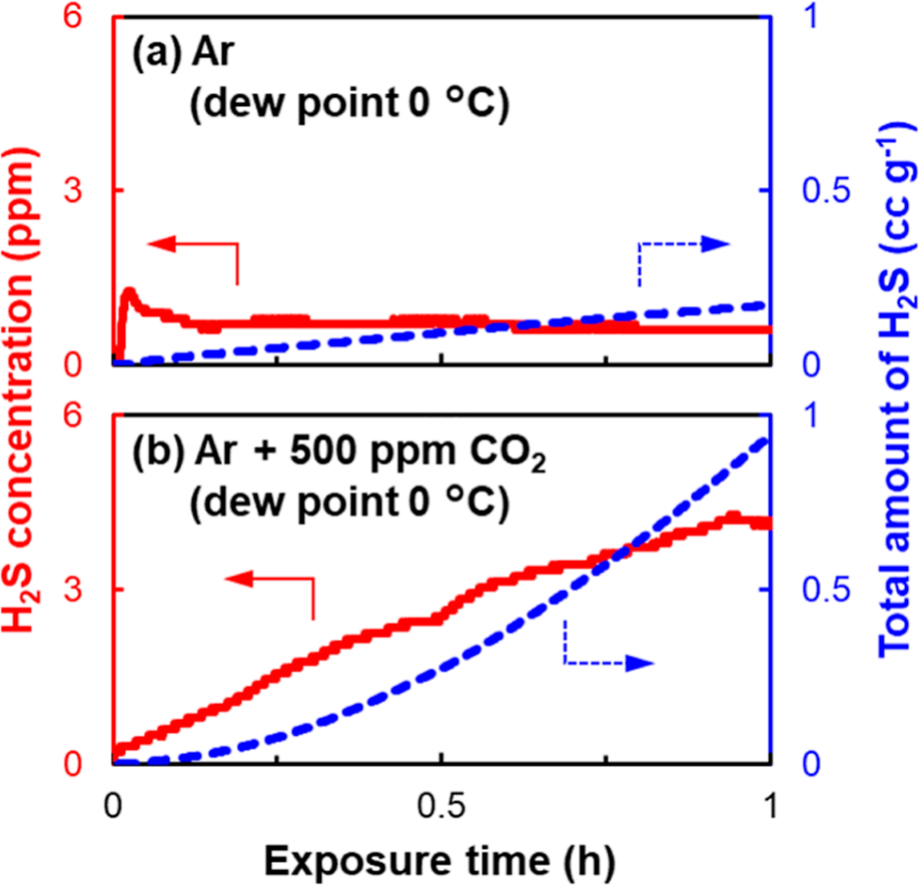
H_2_S gas generation at a dew point of 0 °C in Ar: (a) without CO_2_ and (b) with 500 ppm of CO_2_; H_2_S concentration in gas flow (red, solid line) and total amount (blue, dashed line). The total amount of H_2_S was converted to cc per 1 g of LSS.

Figure 2 shows the concentration of H_2_S gas generation (i.e., the generation rate) and the total amount of H_2_S (cc) per gram of LSS (cc g^–1^). In the case without CO_2_, the generation rate of H_2_S gas was a constant value of ∼0.6 ppm, and the total amount after 1 h exposure was 0.17 cc g^–1^. The order of the total amount of H_2_S gas was roughly the same as previously reported results.^19,20^ By contrast, in the case of CO_2_, the behavior of H_2_S gas generation was obviously different; the generation rate of H_2_S gas increased with exposure time, resulting in a total amount of 0.95 cc g^–1^, which was more than 5 times higher than the case without CO_2_. Here, it should be noted that the amount of H_2_S per LSS is lower than that per typical Li_3_PS_4_ and argyrodite-structured Li_6_PS_5_Cl.^11,12,19^ Each H_2_S amount of 0.17 and 0.95 cc g^–1^ corresponds to merely <0.4 and 2 Å from the particle surface, respectively, when calculated geometrically under the assumption that one H_2_S molecule is generated from one SnS_4_^4–^ unit. The estimated deterioration thickness also indicates that LSS has a remarkably high moisture durability.

**Figure 3:**
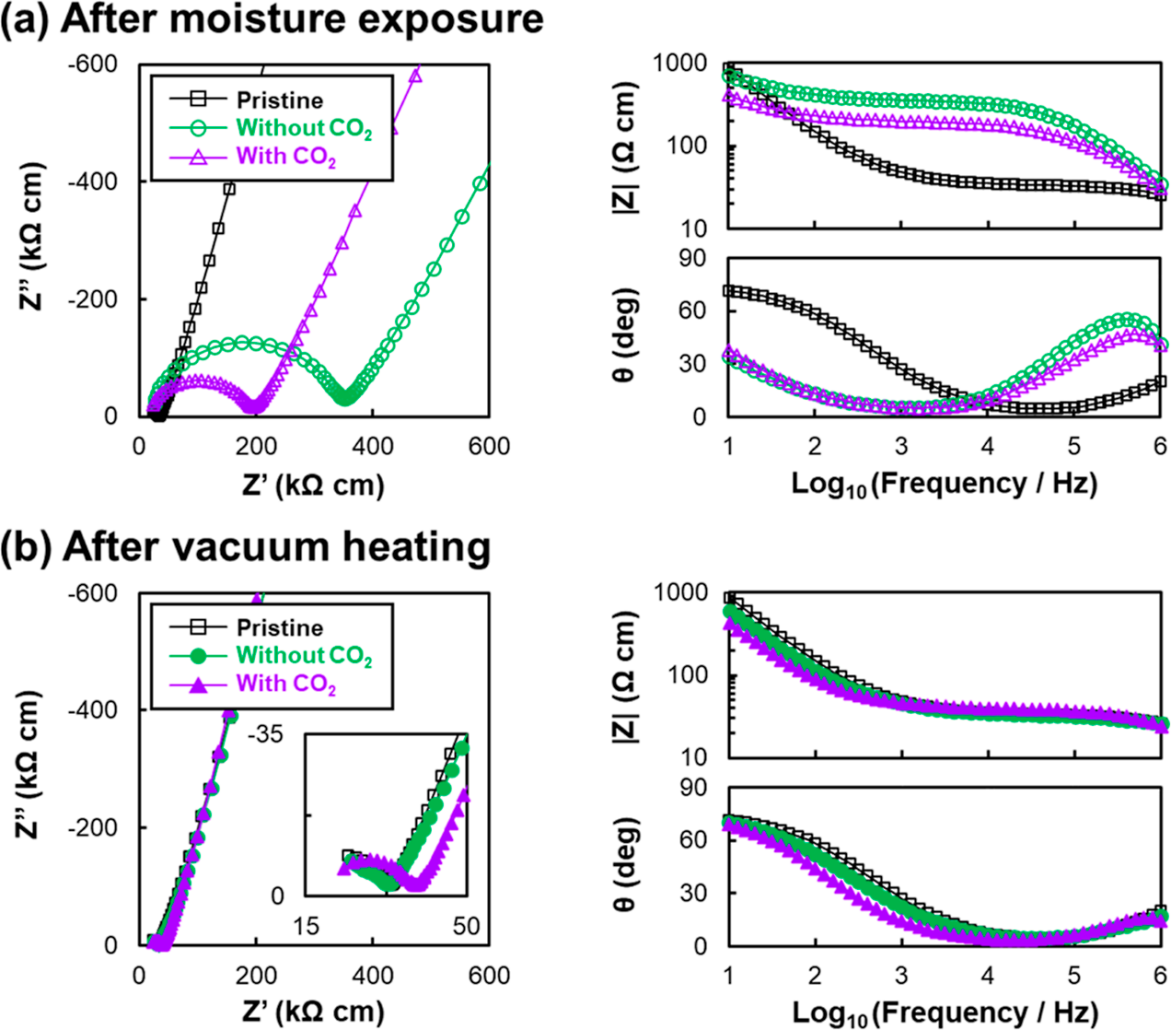
Nyquist and Bode plots of EIS data: (a) after moisture exposure and (b) after vacuum heating. Pristine (black, square), without CO_2_ (green, circle), and with CO_2_ (purple, triangle). Plots describe normalized impedance by pellet thickness and electrode area (unit of kΩ cm). The inset of Figure 3b is a magnified view in a higher-frequency region.

The EIS data shown in Figure 3a reveal an increase in impedance compared to pristine in either case of moisture exposure with and without CO_2_. In other words, lithium ionic conductivity decreased upon moisture exposure with and without CO_2_. Notably, the decrease in lithium ionic conductivity was smaller for the case without CO_2_, although the total amount of H_2_S gas was smaller than that for the case with CO_2_. This result indicates that the decrease in lithium ionic conductivity was not solely caused by the decomposition of the LSS structure with H_2_S desorption. The discrepancy between the total amount of generated H_2_S and the retention of lithium ionic conductivity suggests that the respective deterioration modes in the case with and without CO_2_ can be different from each other.

Figure 3b shows the EIS data of vacuum-heated samples after both moisture exposures compared to the pristine sample. It reveals that the deteriorated lithium ionic conductivities have been significantly recovered and returned to almost the same value as the pristine sample. However, in more detail, there were differences in the spectral shape in higher-frequency regions. A small semicircle remained in the EIS data after vacuum heating the sample exposed to moisture and CO_2_. This suggests that the surface chemical state after vacuum heating differs from the pristine state. The semicircle in the higher-frequency region is attributed to surface species. In fact, EIS studies^11,27,32^ revealed that the impedance components of some sulfide SEs increase due to surface degradation species that result from moisture exposure. Therefore, the incomplete recovery in EIS for the sample exposed to moisture and CO_2_ indicates that irreversible reactions occurred at the SE surface. Table 1 summarizes the total amount of H_2_S gas, lithium ionic conductivity, and the retention value of lithium ionic conductivity for each sample. To elucidate in detail both deterioration and recovery mechanisms upon moisture exposure with and without CO_2_, XRD and Raman spectroscopy analyses were conducted.

**Table 1:** Total Amount of H_2_S upon Moisture Exposure to Ar Gas at a Dew Point of 0 °C for 1 h, Lithium Ionic Conductivity, and Retention Value of Lithium Ionic Conductivity for Each Sample

		without CO_2_	with CO_2_
total amount of H_2_S (cc g^–1^)		0.17	0.95
lithium ionic conductivity (S cm^–1^)/retention	pristine	2.86 × 10^–5^/(100%)	
	after moisture exposure	2.78 × 10^–6^/9.7%	4.72 × 10^–6^/17%
	after vacuum heating	2.83 × 10^–5^/99%	2.44 × 10^–5^/85%

**Figure 4:**
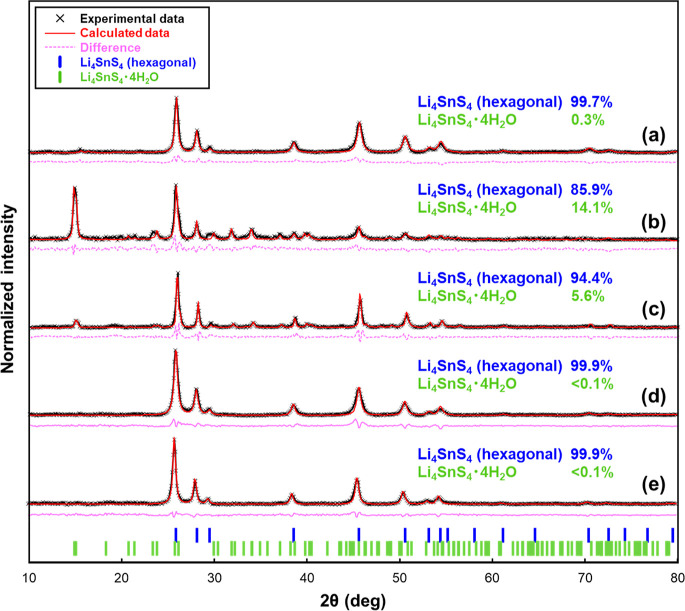
Diffraction pattern analysis: (a) pristine,^27^ (b) moisture-exposed without CO_2_^27^ and (c) with CO_2_, and (d) vacuum-heated after exposure to moisture without CO_2_ and (e) with CO_2_. Experimental data (black, cross mark), calculated data (red, solid line), difference between experimental and calculated data (pink, dashed line), vertical bars at the bottom corresponding to hexagonal Li_4_SnS_4_ (blue) and Li_4_SnS_4_·4H_2_O (light green) crystal structures, respectively. Each percentage value indicates the mol % ratio.

Figure 4 shows the diffraction patterns of samples before and after moisture exposure with and without CO_2_ and after vacuum-heated samples. The pristine LSS has a hexagonal single phase as previously reported (Figure 4a).^19,20,28^ In the case of moisture exposure without CO_2_ (Figure 4b), several new peaks appeared, representing peaks at approximately 14.8, 23.8, 31.9, and 34.1°, among others. The diffraction pattern agrees with that of the hydrate Li_4_SnS_4_·4H_2_O crystal reported by Kimura et al., indicating a change in crystal structure due to hydration upon moisture exposure.^25^ By contrast, despite being exposed to the same amount of H_2_O, the diffraction pattern in the case with CO_2_ has much lower-intensity peaks for hydrate Li_4_SnS_4_·4H_2_O, and the hexagonal phase remained more pronounced (Figure 4c). The molecular composition ratios of Li_4_SnS_4_·4H_2_O determined from quantitative analysis for both exposure conditions with and without CO_2_ are approximately 14.1 and 5.6 mol %, respectively. In either case, the molar ratios of the hydrates are small, suggesting that the hydration reaction occurs only on the surface. These values correspond to hydrate layer thicknesses of approximately 25 and 9.6 nm from the particle surface, which are very small compared with the average SE particle size of approximately 1 μm. Furthermore, in both vacuum-heated samples (Figure 4d,e), patterns of hydrate Li_4_SnS_4_·4H_2_O have completely disappeared and returned to the hexagonal phase. Besides, carbonates, such as Li_2_CO_3_, are known to be deteriorating components associated with CO_2_ on the sulfide SE surface.^12,14,29^ In fact, a nanocoating method has been proposed for sulfide SE Li_6_PS_5_Cl particles, utilizing a surface reaction with CO_2_ gas.^33^ However, no distinct peak corresponding to Li_2_CO_3_^34,35^ is observed, possibly owing to the limitation of XRD as a bulk analysis technique.

**Figure 5:**
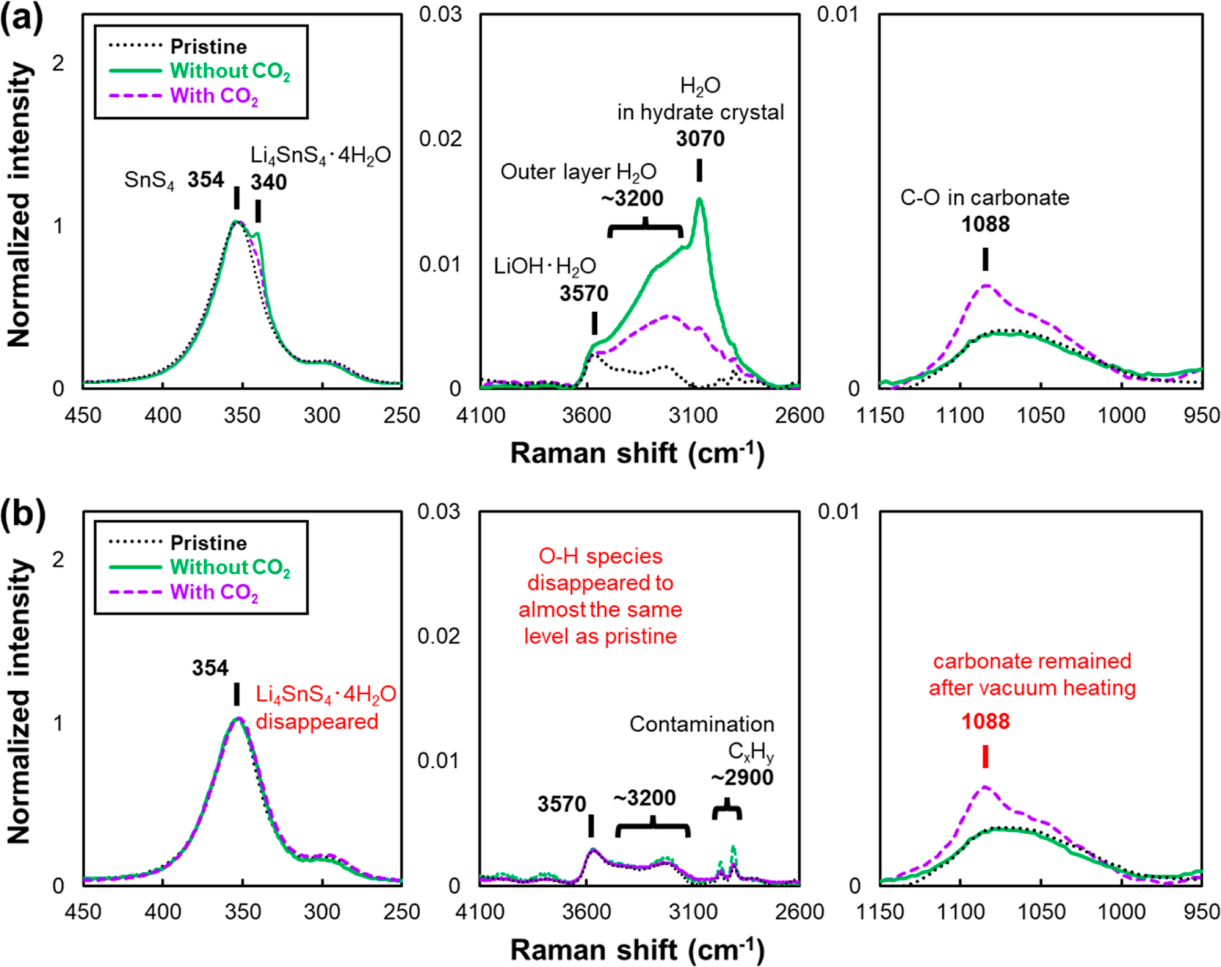
Raman spectra in each region of pristine (black, dotted line), without CO_2_ (green, solid line), and with CO_2_ (purple, dashed line): (a) after moisture exposure^27^ and (b) after vacuum heating.

Surface analysis is required in addition to bulk analysis by XRD, because the hydration reaction of SE upon moisture exposure is considered to proceed gradually from the SE surface, as mentioned above. To analyze the surface while remaining in its hydrate state after moisture exposure, Raman spectroscopy was adopted rather than general surface analyses such as X-ray photoelectron spectroscopy (XPS) and secondary ion mass spectroscopy (SIMS). Yamamoto et al. also conducted Raman spectroscopy as a surface-sensitive measurement used to evaluate the reaction progress of the liquid-phase synthesis of Li_3_PS_4_ by combining with XRD.^36^ Some studies have reported surface analysis for sulfide SE before and after moisture exposure.^12,14^ The analytical depth of Raman spectroscopy in this study is estimated to be several hundred nanometers, although the refractive index and reflectance of the SE are required to calculate the exact numerical value; for example, in the case of a Si wafer, it is approximately 500–600 nm.^36−38^ Raman spectra in the regions of the Sn–S, O–H, and C–O vibrations are shown in Figure 5a. In the region of the Sn–S vibration, the main peak at the 354 cm^–1^ unit and the small subpeak at 300 cm^–1^, corresponding to the SnS_4_^4–^ unit, are observed in the pristine sample. This spectral shape of the pristine sample is consistent with the LSS synthesized by mechanical milling, as previously reported.^19^ After moisture exposure, a new small peak appears at a wavenumber lower than that of the main peak in both spectra of the exposed samples. The new peak at 340 cm^–1^ is assigned to hydrate Li_4_SnS_4_·4H_2_O.^25^ The intensity of the hydrate peak is stronger without CO_2_ than that with CO_2_. The hydrate peaks of both moisture-exposed samples disappeared after vacuum heating. The changes in the O–H stretching vibration region naturally align with the abovementioned change in the Sn–S vibration. Focusing on the O–H stretching vibration mode of H_2_O molecules is powerful for analyzing the chemical and physical states of H_2_O.^12,13,39−45^ Although the Raman scattering sensitivity of the stretching vibration is not very high, a small change is observed. The slight peaks around 2950 cm^–1^ may be attributable to hydrocarbon contamination (C_*x*_H_*y*_) in the Raman spectroscopy measurement cell.^12^ In the O–H stretching vibration region of pristine samples, almost no Raman signals are detected, but a broad band around 3200 cm^–1^ and a small peak at 3570 cm^–1^, assigned to LiOH·H_2_O and the adsorbed outer layer H_2_O by the hydrogen bond network,^12,40^ are observed. These may be very slight residuals due to the aqueous synthesis method or an unavoidable product by storage in a glovebox. Upon moisture exposure without CO_2_, a new peak around 3070 cm^–1^ appears in addition to the increase in the intensity of the broad band around 3200 cm^–1^. Several studies on hydrates have reported that the vibrational peak of the H_2_O fixed in some crystal structures appears around 3100 cm^–1^.^45−49^ Therefore, the new peak is assigned to the internal H_2_O molecule in the hydrate Li_4_SnS_4_·4H_2_O crystal structure in the form of LiS_2_(H_2_O)_2_.^25^ Interestingly, despite the same moisture content of the exposure gas, the intensity of both internal H_2_O in LiS_2_(H_2_O)_2_ and outer layer H_2_O is lower in the case with CO_2_ than in the case without CO_2_, which is consistent with the result on Li_4_SnS_4_·4H_2_O generation in the SnS_4_ region.

Besides, the hydrate markers, such as the new peak at 340 cm^–1^ in the Sn–S region and the O–H stretching vibration bands, in both moisture-exposed samples disappear after vacuum heating (Figure 5b). The trend of Raman peak changes in Sn–S and H–O–H vibration related to hydrate Li_4_SnS_4_·4H_2_O completely agrees with the result of XRD. However, a slight peak of symmetric stretching vibration of C–O in carbonates at ∼1090 cm^–1^^34,50,51^ is observed by Raman spectroscopy only in the sample with CO_2_ and remained even after vacuum heating. These results of XRD and Raman spectroscopy measurements reveal that the introduction of CO_2_ with moisture has resulted in the suppression of hydrate Li_4_SnS_4_·4H_2_O generation and the formation of a small amount of irreversible carbonate species on the SE surface. The contrasting surface states propose different deterioration and recovery mechanisms and support the different behaviors of lithium ion conductivity for the cases with and without CO_2_.

**Figure 6:**
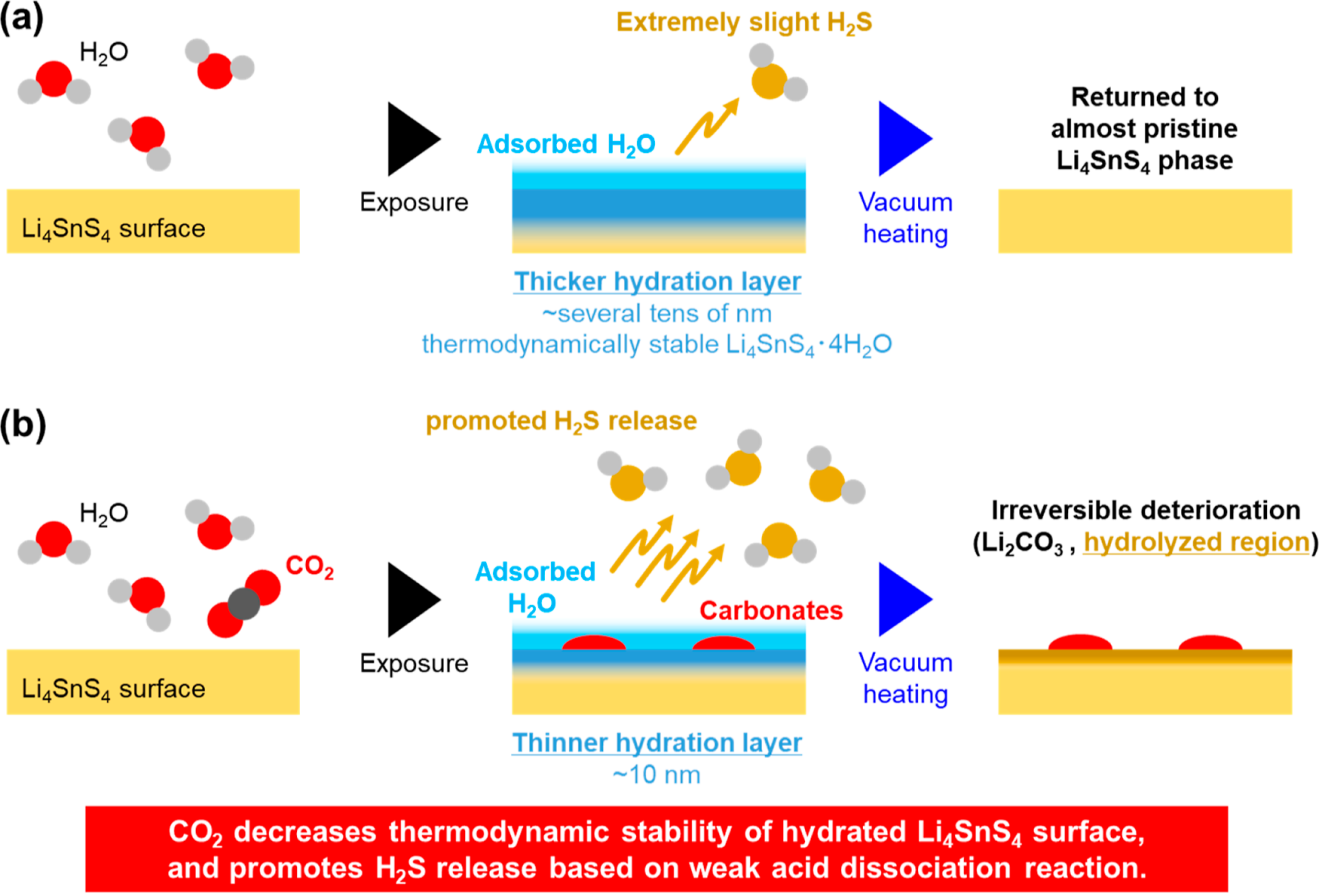
Schematic illustrations of the Li_4_SnS_4_ surface upon moisture exposure (a) without and (b) with CO_2_. The coexistence of CO_2_ decreases the thermodynamic stability of Li_4_SnS_4_·4H_2_O to promote H_2_S release based on a weak acid dissociation reaction and carbonate species generation on the surface, leading to a decrease in lithium ionic conductivity.

The deterioration and recovery mechanism upon moisture exposure without CO_2_ is simply explained as follows (Figure 6a): The hydrate Li_4_SnS_4_·4H_2_O is generated on the LSS surface owing to H_2_O attack. The hydrate is thermodynamically stable owing to S sharing between the LiS_2_(H_2_O)_2_ tetrahedron and the SnS_4_^4–^ tetrahedron.^25^ As the hydration progresses, more hydrate is detected via XRD. However, if the frequency of H_2_O attacks on the LSS surface exceeds the rate at which it penetrates into the internal SE bulk region, then thermodynamic stability is disrupted, resulting in a slight amount of H_2_S gas generation. Kaib et al. previously proposed the crystal structure of Li_4_SnS_4_·13H_2_O as a hydrate with a larger number of H_2_O molecules. It exhibits a NaCl-type crystal structure, consisting of SnS_4_^4–^ tetrahedral anion unit and [Li_4_(H_2_O)_13_]^4+^ hydrate complex cation units, which are connected through hydrogen bonds.^52^ Although Li_4_SnS_4_·4H_2_O and Li_4_SnS_4_·13H_2_O are presumed to be thermodynamically stable, if an intermediate state between them as a transition state is formed, thermodynamical stability decreases for the S-containing units such as Li_2_S and SnS_4_^4–^, leading to a slight amount of H_2_S generation as mentioned above. Additionally, the outer layer H_2_O would form through a hydrogen bond network from the triggered sites such as LiOH·H_2_O and Li_4_SnS_4_·4H_2_O or physical adsorption.^12,13,53^ As a result, hydrate Li_4_SnS_4_·4H_2_O (∼10^–9^ S cm^–1^^25^) and outer layer H_2_O decrease lithium ionic conductivity by inhibiting the conduction on the SE surface. However, these hydrated species reversibly return to the LSS by dehydration and desorption with vacuum heat treatment. As mentioned in the earlier paragraph, the thickness calculated for the H_2_S-released layer was extremely thin (<0.4 and 2 Å), and the lithium ionic conductivity was recovered to almost the same level as the pristine state.

For the moisture exposure with CO_2_, we propose that the deterioration mechanism upon exposure to moisture and CO_2_ involves a “concerted influence” (Figure 6b). Comparing the dissociation constants of CO_2_ (H_2_CO_3_) and H_2_S, it is well-known that CO_2_ has a smaller dissociation constant.^54−56^ In other words, CO_2_ is more acidic than H_2_S. Therefore, the presence of CO_2_ in H_2_O is considered to facilitate the release of H_2_S through a weak-acid dissociation reaction, which is a fundamental chemical reaction. The coordination reaction of CO_2_ is more dominant. As a result, the hydrated species decrease and the surface undergoes hydrolysis, leading to the release of H_2_S. Simultaneously, a small amount of carbonates is also formed on the SE surface. Although the hydrated species can reversibly return to LSS through vacuum heating, the hydrolyzed layer (a few nanometers) caused by H_2_S releasing and carbonate species formation slightly decreases lithium ionic conductivity, resulting in irreversible deterioration on the SE surface even after surface dehydration by vacuum heating occurs. Therefore, the “concerted influence” resulting from the coexistence of H_2_O and CO_2_ based on a weak-acid dissociation reaction reduces the thermodynamic stability of Li_4_SnS_4_·4H_2_O, promoting H_2_S release and carbonate species formation on the surface, ultimately leading to a decrease in lithium ionic conductivity. Here, it should be emphasized that carbonates, such as Li_2_CO_3_, may not promote H_2_S generation but may be merely byproducts resulting from the addition of CO_2_, which should be elucidated in detail in the future. The concerted influence of H_2_O and CO_2_ suggests a new metric that should be considered in the battery manufacturing process. Additionally, the impact of SE surface degradation on battery performance, which has been partially reported,^33,57−59^ is one of the future research targets.

## Conclusions

In this study, we investigated the surface hydrolysis deterioration when a sulfide SE, LSS, was exposed to Ar gas at a dew point of 0 °C, both with and without 500 ppm of CO_2_. The amount of H_2_S gas generation varied depending on the presence or absence of CO_2_, despite being exposed to the same amount of H_2_O. In the presence of CO_2_, H_2_S gas generation increased by more than five times. However, the lithium ion conductivities significantly decreased after moisture exposure, regardless of the presence or absence of CO_2_. XRD and Raman spectroscopy analyses indicated that the deterioration mechanisms differed noticeably between the two cases. Without CO_2_, the thermodynamically stable hydrate Li_4_SnS_4_·4H_2_O formed on the surface, resulting in minimal H_2_S gas generation and demonstrating excellent reversibility through dehydration with vacuum heating. By contrast, in the presence of CO_2_, a weak acid dissociation reaction promoted the generation of H_2_S and carbonate species on the surface, leading to a decrease in the lithium ionic conductivity. The hydrolyzed species also reduced the reversibility upon dehydration. Further research is needed to elucidate intermediate reactions that occur when H_2_O and CO_2_ coexist; moreover, the relationship between atmospheric conditions, such as dew point and gas species, as well as the influence of SE species itself should be explored in more detail. This finding of the “concerted influence” of H_2_O and CO_2_ provides valuable insights into material development and future implementation.
